# Contour features predict valence and threat judgements in scenes

**DOI:** 10.1038/s41598-021-99044-y

**Published:** 2021-09-30

**Authors:** Claudia Damiano, Dirk B. Walther, William A. Cunningham

**Affiliations:** 1 Department of Brain and Cognition, KU Leuven, Tiensestraat 102 - box 3711, 3000 Leuven, Belgium; 2 Department of Psychology, University of Toronto, Toronto, Canada

**Keywords:** Human behaviour, Perception

## Abstract

Quickly scanning an environment to determine relative threat is an essential part of survival. Scene gist extracted rapidly from the environment may help people detect threats. Here, we probed this link between emotional judgements and features of visual scenes. We first extracted curvature, length, and orientation statistics of all images in the International Affective Picture System image set and related them to emotional valence scores. Images containing angular contours were rated as negative, and images containing long contours as positive. We then composed new abstract line drawings with specific combinations of length, angularity, and orientation values and asked participants to rate them as positive or negative, and as safe or threatening. Smooth, long, horizontal contour scenes were rated as positive/safe, while short angular contour scenes were rated as negative/threatening. Our work shows that particular combinations of image features help people make judgements about potential threat in the environment.

## Introduction

It is fundamental to survival to be able to accurately perceive scenes, i.e., the real-world environments in which people find themselves every second of every day, and to safely and rapidly make decisions on how to act (e.g., to approach or to avoid the scene)^1–3^. How do people achieve this basic yet essential behaviour? Here, we explore this question in the visual domain, specifically investigating which visual features play a role, and how.

As these judgements and decisions happen extremely rapidly, it has been proposed that low- and mid-level features, such as low spatial frequencies^4^, curvilinearity^5^, and angularity^6,7^ influence rapid threat detection and judgements of valence. The processing of visual features related to negative or threatening stimuli in particular, has been thought to drive evolution such that animals, including humans, have evolved to quickly detect potential danger^2,5^. For example, angularity plays a large role in ratings of “liking” and threat, such that semantically neutral objects with angular contours are liked less and judged as more threatening than objects with smooth contours^6^. The authors suggest that angularity might be one such feature that we have evolved to detect as threatening, because it is highly correlated with negative stimuli. Specifically, angularity is related to dangerous stimuli such as sharp teeth or thorns, or a threatening human face^8^, increasing amygdala activity^9,10^ and thus triggering a negative bias. The preference for smooth over angular contours has also been studied and found in non-human primates^11^ and human infants^12^, further supporting an evolutionary account.

While we have learned much from these studies, the results are inherently limited in their ecological relevance and generalizability due to the fact that they used simple geometric shapes or isolated objects against a blank background. In daily life, decisions to approach or avoid a location likely do not rely on a sole object or feature but rather a combination of features representing the state of the environment *as a whole*. Theories of visual perception, such as the Reverse Hierarchy Theory^13^ and the coarse-to-fine hypothesis^14^ suggest that scene perception first allows for a general understanding of the visual environment (i.e., the scene gist), followed by more detailed perception of local objects or features. Indeed, previous work has shown that global scene layout gives enough information for people to accurately identify a scene’s category. For example, beaches have a larger proportion of long horizontal lines (i.e., the horizon) than other scene categories^15^, and are considered more “open” environments^14^. Thus, in this study, we measure and manipulate the physical characteristics of scenes that may allow people to make rapid judgements, not only about a scene’s category, but also about the relative threat and emotional valence of the scene.

As discussed above, understanding the scene context in order to make rapid threat judgements may be even more basic than object perception, since the gist of a scene is thought to be processed more quickly than the individual objects within the scene^13,14^. With that in mind, one could assume that it requires extra processing effort to be aware of the individual objects within a scene. In daily life, it makes little sense to be hypervigilant for dangerous objects at all times. This would use up precious cognitive resources. Rather, a sequential approach whereby emotional appraisals arise from a series of stimulus evaluation checks^16^ would be much more advantageous. In other words, a perceiver simply needs clues to the environmental context in order to determine how to proceed with cognitive processing, such as choosing to avoid the scene, or expressing a particular emotional response such as fear in order to enhance sensory processing^17^.

Despite the obvious fact that perceivers are always *in* a scene^18^, the visual features of scenes that may provide these clues needed for emotional appraisals and decision making have barely been explored. To date, only a handful of studies have investigated the impact of smooth vs. angular contours on judgements of beauty and approach-avoid decisions in scenes or scene-like images, using indoor rooms^19^ and building façades^20^ as stimuli. Thus, we seek to further understand how a subset of contour features influence people’s judgements of positive and negative valence in complex real-world scenes. For this, we use two approaches. The first is to extract contour features from photographs in the International Affective Picture System (IAPS), which is a large database of emotionally-evocative colour photographs with existing valence ratings, spanning a wide range of image content – from cute babies and animals, to tragic events and natural disasters, etc. Importantly, the images consist of objects embedded within scenes, as well as landscape images, rather than individual objects removed from their scene context. The second approach is to create our own abstract scene images using combinations of contour features and to collect ratings of the valence and threat level of these images.

Some researchers have used the IAPS image set to relate valence ratings to certain visual features, such as brightness^21^, colour^22^, spatial frequency content^23^, and complexity measures such as global symmetry, edge density, and self-similarity, among others^24^. However, we know that the more significant features for scene perception include the contour features that cue scene structure and layout, such as length^25^, junctions^15,26^, and local symmetry^27^. Additionally, recent work has found that high spatial frequency information conveys scene content better than low spatial frequency information when contrast is equated across images^28,29^. Thus, here we chose to focus on features that could be extracted from high spatial frequency edges of scene images. These types of contour features have not been studied in the context of emotionally evocative photographs, likely due to the fact that it is difficult to extract contour information from photographs.

Our method uses a simple yet effective manipulation – line drawings traced from photographs – which allow us to directly measure contour features within the IAPS image set. We explore the link between contour features and valence ratings in photographs of real-world scenes. Once we identify the features influencing emotional judgements of scenes, we also explore whether combinations of these features influence emotional judgements, absent of explicit semantic content. By creating “abstract” scenes out of these contour features, we seek to determine whether a combination of orientation, length, and angularity statistics in scenes have a direct influence on people’s valence and threat judgements.

## Results

### Experiment 1

**Figure 1:**
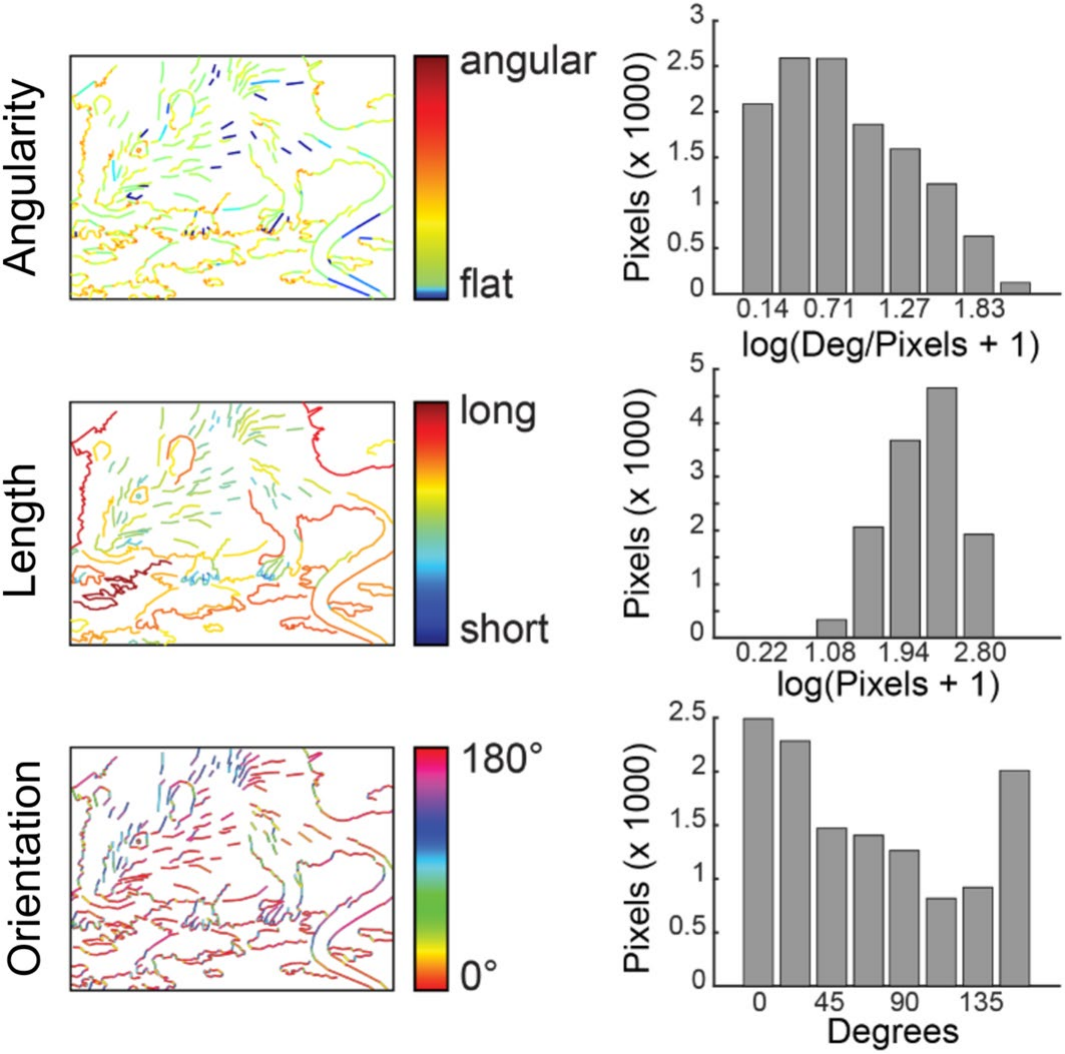
Feature histograms. Depictions of the distribution of feature values on the contours, with the corresponding feature histograms, from one example line drawing (IAPS image #1280).

In order to first assess the influence of contour features on valence ratings of a set of photographs, we performed a multiple linear regression analysis, predicting valence ratings of each IAPS photograph from the contour features of the traced IAPS line drawings. The line drawings were produced by artists at the Lotus Hill Research Institute (People’s Republic of China) by tracing the original IAPS colour photographs using a custom graphical user interface. The artists were instructed to trace all important lines so that if a human observer were to look at the line drawings, they would be able to recognize the image (i.e., the scene being depicted and the objects within the scene). Using these line drawings, contour properties (orientation, length, and angularity) were computed from the geometrical information of the line drawings. See Fig. 1 for an example image and its feature histograms. Each histogram was computed in eight bins, each bin corresponding to a particular range of values. All feature values included in the regression were square root transformed to reduce outliers.

The multiple linear regression analysis yielded a significant regression equation (*F*(22,1159) = 2.103, *p* = 0.002), with an *R*^2^ of 0.04. The significant predictors of valence ratings were Angularity 8 (i.e., number of pixels that belong to highly angular contours), *β* = − 0.08, *p* = 0.02, Length 7 (i.e., number of pixels that belong to long contours), *β* = 0.01, *p* = 0.007, and Orientation 5 (i.e., number of pixels that belong to vertical contours), *β* = − 0.03, *p* = 0.001.

**Figure 2:**
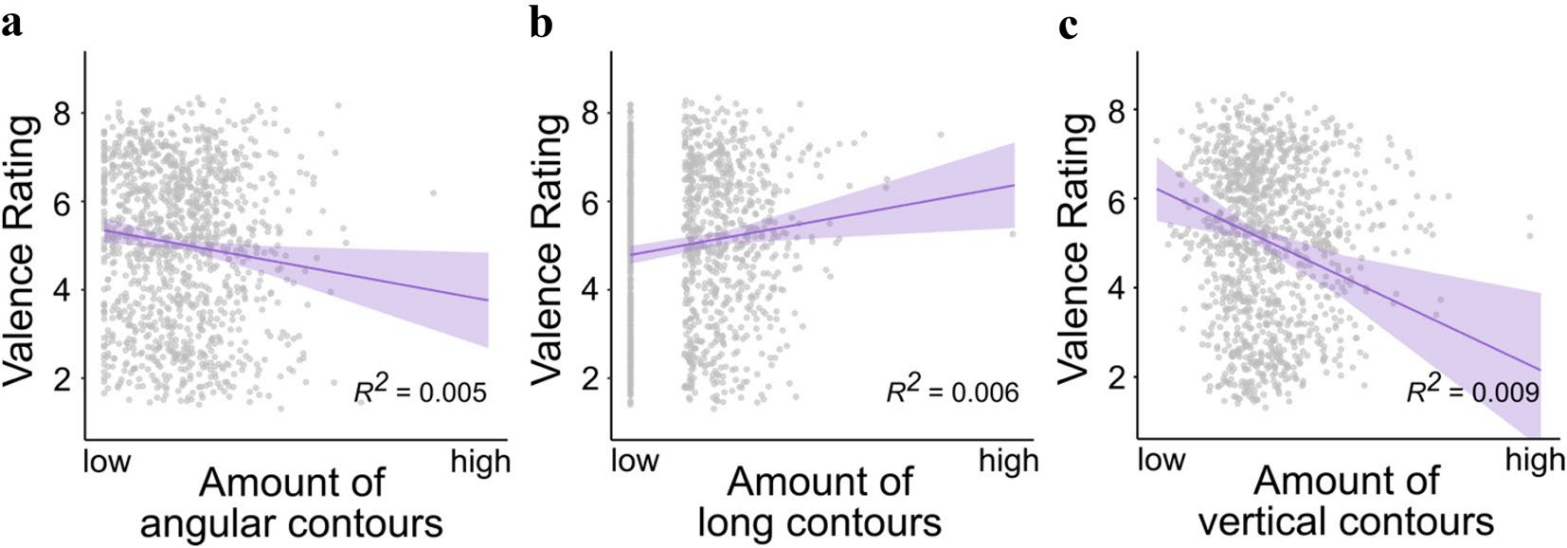
Partial regression plots of the three significant predictors. **a** Average valence ratings of IAPS photographs as a function of the number of pixels belonging to highly angular contours in that image; **b** Average valence ratings as a function of the number of pixels belonging to long contours in that image; **c.** Average valence ratings as a function of the number of pixels belonging to vertical contours in that image. Valence ratings ranged from 1 (“unhappy”) to 9 (“happy). The grey dots in each graph represent individual images. The shaded areas show 95% confidence intervals.

Figure 2a shows that as the number of pixels that belong to angular contours increases, valence ratings decrease. Figure 2b shows that as the number of pixels that belong to long contours increases, valence ratings increase. Finally, Fig. 2c shows that as the number of pixels that belong to vertical contours increases, valence ratings decrease.

The IAPS images are a protected image set and are therefore not allowed to be published in any capacity. However, the table below (see Table 1) contains the one-word descriptions (as provided in the IAPS database) of the top and bottom three images containing the greatest and the least, respectively, of each of the significant features with their valence scores. For example, the image containing the smallest number of pixels that belong to vertical contours) depicts a mountainous landscape, while the image containing the most of that feature is a checkerboard pattern. Note that these are not necessarily the images rated the highest and lowest in valence, though the landscape image did receive a higher valence score (7.29) than the checkerboard pattern image (5.16).

**Table 1:** The image descriptions and valence scores of the three images with the least and three images with the greatest number of pixels corresponding to the significant features shown in Fig. 2.

	Bottom three			Top three		
High angularity	Dog	Coyote	Veiled Woman	Rug	Mutilation	Chicken
	7.01	6.27	5.56	5.06	1.45	6.19
Long lines	Snake	Snake	Snake	Violin	Money	Agate*
	4.26	3.8	3.7	6.5	7.51	5.26
Vertical orientation	Mountains	Abstract Art	Puzzle	Jail	City	Checkerboard
	7.29	4.97	5.4	3.73	6.05	5.16

*Agate is a type of gemstone.

The results support the hypothesis that at least some image features (i.e., contour features) predict valence ratings of photographs. The presence of long contours is positively related to valence ratings, while the presence of vertical contours and angular contours is negatively related to valence. A particular image which contained many high angularity contours depicted a disturbing scene of mutilated and burned corpses. Images containing few high angularity contours depicted more positive stimuli, such as a dog or a beautiful veiled woman. A similar finding is observed when looking at the Orientation feature. Images containing few vertical contours depicted natural landscapes or abstract art, while those high in vertical contours depicted more cluttered scenes such as a city street, or a prison cell with large vertical bars.

A striking finding when looking at individual exemplars of the Length feature (see Table 1) is the fact that the three images with the fewest long contours all depicted snakes. This is somewhat surprising when considering that snakes, in their simplest form, are described as long curvilinear lines^5^. We find that, in these more realistic photographs of snakes, there are actually many short triangular contours, corresponding to the scales of the snake skin. This suggests that researchers may have to rethink their depictions of snakes when studying the visual features that influence snake detection. By focusing on the rapid detection of curvilinearity as a proxy for rapid snake detection, researchers may be inadvertently simply showing a fluency effect (i.e., that smooth curves are processed more quickly than sharp angles)^7,30,31^ rather than a snake effect.

Overall, these results reveal that relationships between particular contour features and participants’ valence judgements exist. However, many of the IAPS images are extremely close-up photographs of individual objects, which would suffer from the same problems as stimuli used in past experiments (i.e., isolated objects removed from their scene contexts). In order to test whether the presence of the scene context is an important aspect of the predictive power the contours, we separated the IAPS image set into two rough categories based on the spatial scale of the central object and how much of the scene background was visible. The categories were “close” (i.e., the central object or figure takes up a large portion of the total area, and the scene background is barely or not at all visible) and “far” (i.e., the central objects or figure takes up a smaller portion of the total area and the scene background is visible, or there is no central object or figure present and the scene context is fully visible). There was a total of 456 close images and 707 far images.

We ran separate multiple linear regressions for each image set (close and far) and found that the regression analysis yielded a significant regression equation (*F*(22,684) = 2.675, *p* < 0.001, *R*^2^ = 0.08) for the far images, but not for the close images (*F*(22,433) = 1.436, *p* = 0.09), which confirms our hypothesis that the scene context is important. For the far images, the significant predictors of valence ratings were Length 5 (*β* = 0.03, *p* = 0.03) and 7 (*β* = 0.02, *p* = 0.004) (i.e., number of pixels that belong to medium-long and long contours), with Angularity 8 (*β* = − 0.10, *p* = 0.04) and Orientation 5 (*β* = − 0.03, *p* = 0.02) also playing a role, similarly to the results of the overall model with the full image set.

### Experiment 2

**Figure 3:**
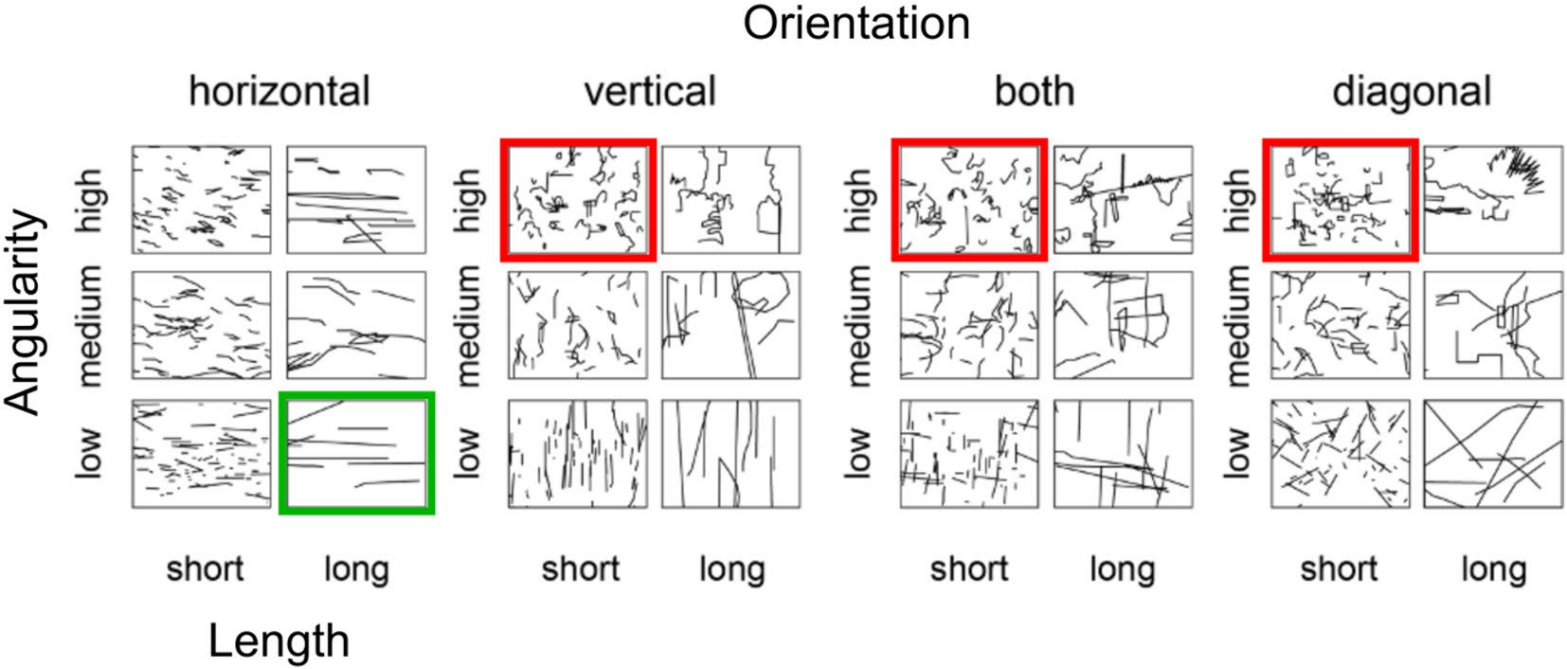
Sample abstract scenes with different combinations of features. One example is shown for each bin. The image outlined on the bottom row is considered the most positive/safe according to the results of Experiment 2. The three images outlined on the top row are considered the most negative/threatening.

The above results reveal that certain contour features predict valence ratings of photographs, and this effect is seemingly driven by the photographs that contain scene background. However, even for such images, the percent of explained variance is still fairly low, and several confounding factors may be driving the significant results. For example, the line drawings were extracted from the photographs via artists’ tracings. It could be the case that artists imposed their own biases when tracing the photographs, highlighting certain contours and ignoring others depending on the emotional valence of the photograph. We do not think it is a problem, since any computational edge detection algorithm would also fall victim to biases depending on its parameters. More importantly, the results found in Experiment 1 may suffer another confound: they are likely biased by the semantic content available in the images, and the fact that, for the most part, there is still a central object of focus in each image. Therefore, in experiments 2a and 2b, we sought to explore the link between visual features and emotions using scene-like stimuli that were not confounded with explicit semantic content or the biases of artists tracing photographs in a potentially biased manner. In two online experiments, participants viewed abstract, content-free line drawings of scenes artificially generated so that they adhere to particular statistics of their visual features (see Fig. 3). On each trial, four such images were displayed at once and participants were asked to rate one image as the most positive and another as the most negative (Experiment 2a; affective valence judgement) or as the most threatening and safe (Experiment 2b; threat judgement).

To assess the influence of contour features on positive and negative judgements of abstract line drawings, we performed a logistic regression (using glmer from the “lme4” package^32^ in R^33^, modelling valence judgements (positive = 1, negative = 0) as a function of Length (short, long), Angularity (low, medium, high), and Orientation (horizontal, vertical, both, diagonal), as well as the interactions between these variables, and trials nested in participants as random effects. The neutral images (i.e., those not selected as positive/safe or negative/threatening) in each trial were not included in the model.

#### Experiment 2a

Using likelihood-ratio tests, as implemented in the afex package^34^ in R, we found main effects of Orientation, χ^2^(3) = 1242.85, p < 0.001, Angularity, χ^2^(2) = 1310.83, p < 0.001, and Length, χ^2^ (1) = 643.38, p < 0.001. These main effects indicate the following: 1) images containing horizontal contours are more likely to be rated as positive than images containing contours of other orientations (i.e., vertical, both horizontal and vertical together, or diagonal contours), which are more likely to be rated as negative; 2) images containing contours with low angularity (i.e., smooth/flat contours) are more likely to be rated as positive than images containing highly angular contours, which are more likely to be rated as negative; and 3) images containing long contours are more likely to be rated as positive than images containing short contours, which are more likely to be rated as negative.

In addition to the main effects, we found a significant interaction between Orientation and Angularity, χ^2^ (6) = 254.59, p < 0.001, where images containing smooth (i.e., low angularity) contours are more likely to be rated as positive than images containing medium and high angularity contours across all orientations, except horizontal. In other words, if the high and medium angularity contours have an average horizontal orientation, those images will be rated as more positive than if the contour orientations are not horizontal. There was also an interaction between Orientation and Length, χ^2^ (3) = 9.76, p = 0.020, where images that contain long contours are more likely to be rated as positive than images containing short contours, but this difference is minimized for images that contain horizontally oriented contours. Finally, there was also a significant interaction between Angularity and Length, χ^2^ (2) = 13.96, p < 0.001, where images that contain long contours are more likely to be rated as positive than images containing short contours, and this difference is slightly larger when the contours have low angularity compared to when they have medium or high angularity.

**Figure 4:**
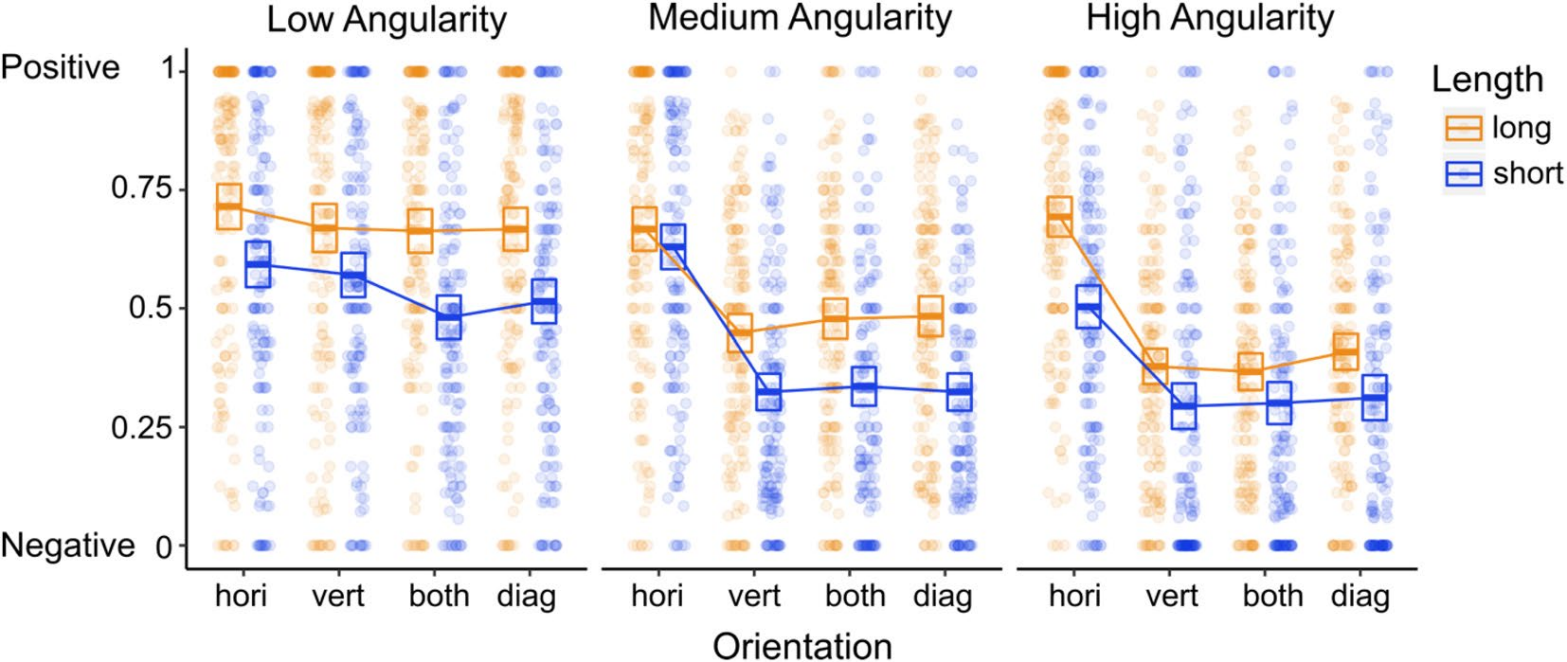
Average positive and negative scores as a function of image contour features. The boxes represent the 95% confidence intervals. The dots represent individual (*N* = 157) average values per condition.

Finally, the three-way interaction was significant, χ^2^ (6) = 57.89, p < 0.001, where the length effect is minimized when contours contain medium angularity, and the orientation effect is minimized when contours contain low angularity (see Fig. 4). According to these findings, an image containing short, highly angular, non-horizontal contours would be most likely to be rated as negative (for example, see Fig. 3 top row, third, fifth, and seventh image from the left, marked in red), while an image containing long, low angularity, horizontal contours would be most likely to be rated as positive (see Fig. 3 bottom row, second from the left, marked in green).

#### Experiment 2b

So far, we have explored these visual features in relation to general valence judgements, rather than threat judgements specifically. Thus, Experiment 2b explicitly asked participants to judge threat and safety. The experiment was identical to Experiment 2a, except that participants were asked to indicate which of the displayed images they found to be the most threatening and which the safest. To assess the influence of contour features on safety and threat judgements of abstract line drawings, we performed another logistic regression, as in Experiment 2a. We modelled threat judgements as a function of Length, Angularity, and Orientation, as well as the interactions between these variables, and trials nested in participants as random effects.

Using likelihood-ratio tests, we found that all main effects and interactions were significant. Firstly, the main effect of Orientation, χ^2^ (3) = 2366.10, p < 0.001, indicates that images containing horizontal contours are more likely to be rated as safe than images containing contours of other orientations (i.e., vertical, both horizontal and vertical together, or diagonal contours), which are more likely to be rated as threatening. The main effect of Angularity, χ^2^ (2) = 1940.16, p < 0.001, indicates that images containing contours with low angularity (i.e., smooth/flat contours) are more likely to be rated as safe than images containing highly angular contours, which are more likely to be rated as threatening. Finally, the main effect of Length, χ^2^ (1) = 4410.36, p < 0.001, indicates that images containing long contours are more likely to be rated as safe than images containing short contours, which are more likely to be rated as threatening.

The significant interaction between Orientation and Angularity, χ^2^ (6) = 466.40, p < 0.001, shows that images containing smooth (i.e., low angularity) contours are more likely to be rated as safe than images containing medium and high angularity contours across all orientations, except horizontal. The interaction between Orientation and Length, χ^2^ (3) = 29.75, p < 0.001, indicates that the effect of orientation is more pronounced when images contain short contours compared to long contours. On the other hand, the interaction between Angularity and Length, χ^2^ (2) = 136.44, p < 0.001, shows that the effect of angularity is more pronounced when images contain long contours compared to short contours.

**Figure 5:**
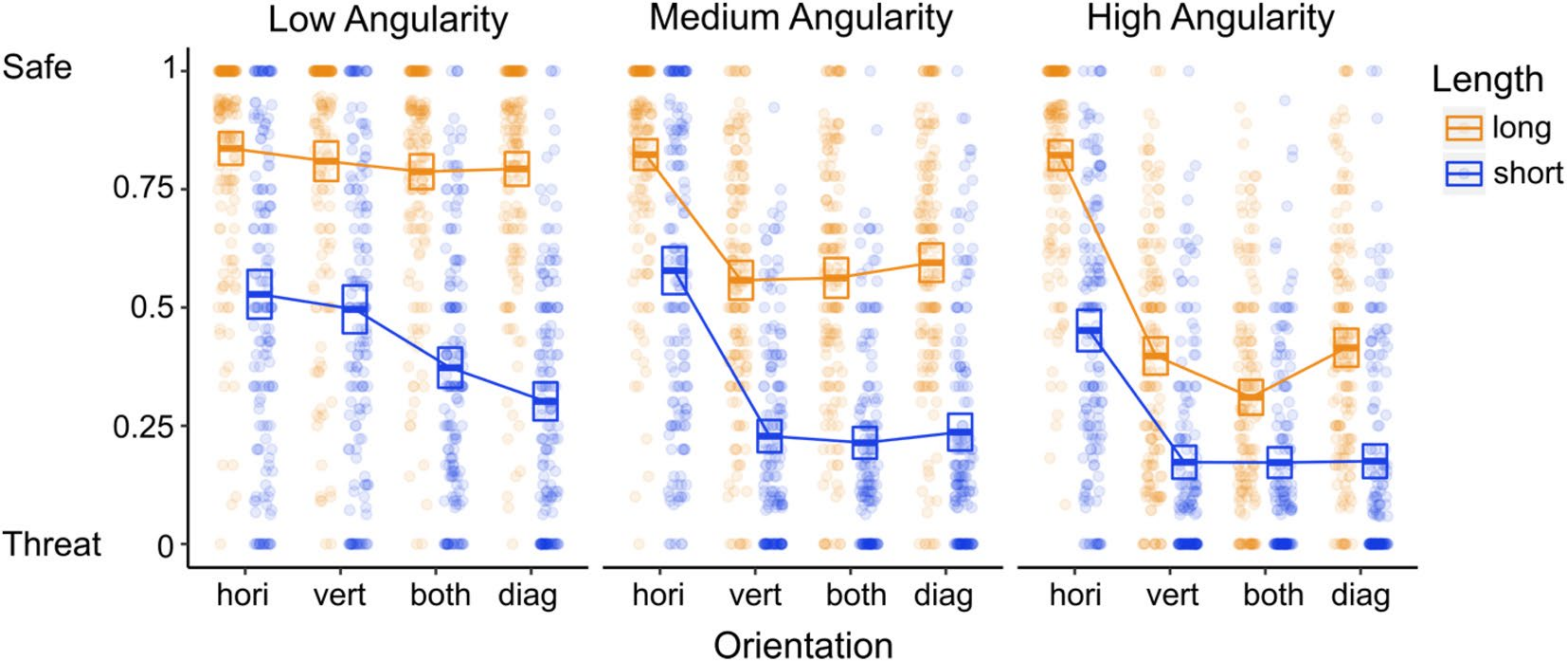
Average safe and threatening scores as a function of image contour features. The boxes represent the 95% confidence intervals. The dots represent individual (*N* = 157) average values per condition.

Finally, there was a significant three-way interaction, χ^2^ (6) = 110.50, p < 0.001, where the length effect is minimized when contours have medium and high angularity and certain average orientations, and the orientation effect is somewhat minimized when contours have a low angularity. Similar to the results of Experiment 2a, these findings indicate that an image containing short, highly angular, non-horizontal contours would be most likely to be rated as threatening, while an image containing long, low angularity, horizontal contours would be most likely to be rated as safe (see Fig. 5).

#### Free responses

Following Experiments 2a and 2b, participants had the opportunity to give free responses to the questions “Which features do you typically associate with positive/safe images?”, and “Which features do you typically associate with negative/threatening images?”. All participants’ written responses can be found on our OSF page (https://osf.io/y3rjm/).

Consistent with the rating results, participants mention features such as “straight” and “smooth” when describing which features they associate with positive or safe images, while using words such as “sharp” and “jagged” when describing features they associate with negative or threatening images. Additionally, *only* in the positive/safe responses do participants describe that images that looked like landscapes or other natural scenes were associated with their decisions. Conversely, “abstract” or “meaningless” are only mentioned in relation to the negative/threat responses, although participants also mentioned negative semantic content, such as violence or danger, in relation to negative/threatening responses. These qualitative responses suggest that the features cueing scene structure (long horizontal lines, e.g., the horizon line) are related to the same features that influence positive valence and safety judgements, while the features that cue danger (sharp jagged lines, e.g., thorns or rocks), or that are more ambiguous, influence negative valence and threat judgements. However, another possibility is that participants simply make aesthetic judgements, which they then map on to the positive/safe and negative/threatening responses. Indeed, in Experiment 2a, a small subset of participants mentioned that they based their decisions on which images looked beautiful/pleasant vs. ugly/unpleasant. No participants mentioned beauty or aesthetic pleasure in the free responses of Experiment 2b, suggesting that participants only consider aesthetic appeal when making a general valence judgement and not when specifically judging threat level only, though we cannot fully rule this out.

## Discussion

To summarize, we used an exploratory analysis along with two additional studies using causal stimulus manipulations to show that several contour features are related to emotional appraisals of valence and threat in scenes. Our exploratory analysis (Experiment 1) revealed that the length, orientation, and angularity of contour features indeed relate to participants’ valence judgements of photographs. In Experiments 2a and 2b, we used abstract stimuli that did not contain explicit semantic content in order to explore the relationship between visual features and emotions in a more controlled manner. We found that combinations of contour features (i.e., angularity, length, and orientation), which are important for the rapid perception of scenes, all played a role in influencing people’s judgements of valence, and even more so, of threat, in visual scenes. Our work expands on previous findings regarding the link between angularity and affective judgements and highlights the importance of examining combinations of visual features within the context of scene perception.

Firstly, our finding that angularity is related to negative valence is consistent with previous work using isolated objects, which showed that people preferred objects^6^ or simple geometric shapes^7,35^ with smooth contours over those with angular contours. In other words, sharpness is bad. The common argument for why this is the case is that humans (and other animals) have evolved to quickly detect certain features that have generally been related to dangerous or threatening stimuli. For example, the Snake Detection Theory states that humans and non-human primates have evolved to automatically detect the presence of snakes and react with an automatic fear response^2,36,37^ because it has been evolutionarily important to avoid snakes. This is the same argument used to explain the reason people generally rate angular shapes or objects as more threatening and negative than smooth shapes or objects. It has been suggested that angularity is a cue to dangerous stimuli such as a threatening face, or thorns and sharp teeth that, if encountered, would hinder survival^6,8,9^. Thus, humans must surely have developed a mechanism to detect such dangerous stimuli rapidly and automatically.

There is one striking issue when taking all of these studies together – snakes are often described as long, smooth, and curvilinear, not angular. Human faces are generally rounded, with their features becoming angular to convey negative emotions^8^. Being able to see the “sharp” part of a snake (its fangs, or the pattern of its skin) is scale dependent. If a person were to encounter snake fangs, or to see the furrowed brow of an angry threatening person, it would mean that they were already very close to the threat, and it may be too late for a behavioral response. Importantly, in our current study, we found that certain *combinations* of length, orientation, and angularity features influence valence and threat judgements, rather than curvature or angularity alone. Thus, we propose a slight modification to the theory above. That is, in complex visual scenes (i.e., settings that animals encounter every day), the ability to accurately understand the scene is the driving force behind valence and threat judgements, and other affective judgements in general.

In fact, theories of aesthetic pleasure suggest that processing fluency is a driving factor in aesthetic appreciation^38–40^. Factors that are known to increase processing fluency, such as the Gestalt principles of symmetry and other visual regularities that allow for fluent figure-ground segmentation, have been shown to be positively related to the aesthetic appreciation of objects^39^ and scenes^41^. Interestingly, an evolutionary account has also been proposed for the aesthetic appreciation of visual stimuli in relation to processing fluency. This account suggests that processing fluency plays a role in aesthetic appreciation specifically because of its relationship to threat detection and our ancestors’ ability to find food and avoid predators^42,43^. These theories posit that aesthetic appreciation (of natural scenes) arose from the ability to accurately perceive one’s environment and anticipate danger in order to increase chances of survival. Our work is consistent with this hypothesis, since the very features that allow for rapid scene perception^15^ are those that predict safety and positive valence. More generally, appraisal theories of emotion state that emotions are adaptive responses which are beneficial to an animal’s well-being^16,44^. Thus, while we have focused on threat detection in the current study, it is certainly not the only emotional appraisal that may be related to visual contour features of scenes.

For both theories of threat detection and of aesthetic pleasure of natural scenes, the ability to discriminate a potential threat from a potential food source, for example, seems to be imperative. Scenes in which the entire environment is clearly in view are deemed the most pleasant^45,46^ and the safest^47^ because there exists a clear view of a threat, should one arise. These types of environments are defined by an open setting with a clear horizon and not much else in view, and would be described as having long smooth horizontal contours. In fact, long horizontal contours are directly related to judgements of openness and depth in natural scenes, with horizon lines clearly cueing open, large-scale, environments^48^. These combinations of features are exactly what we found to be related to participants’ judgements of positive valence and safety. On the other hand, an environment containing many shrubs and bushes, or tall grass, would hide the potential threat. Scenes like these would contain many short angular edges that make up the grasses and leaves. The potential for danger is deemed too great, and thus these scenes are judged as negative and threatening because of their ambiguous or mysterious nature.

Of course, this theory cannot account for all judgements of valence in an environment. There are some cases in which an open environment may hide potential danger, such as a shark swimming below the ocean’s surface, while an environment containing tall shrubs may allow people to hide from danger. Affective judgements or decisions to approach or avoid an environment would have also relied on other factors, such as an imminent need for food or shelter. Further work will be needed to refine the theory and to determine the independent contributions of visual and cognitive factors on different types of affective judgements (e.g., threat detection, aesthetic preferences, general valence) of scenes. For now, we focus on the idea that, from a purely visual perspective, it is likely much easier to perceive a potential threat in an open environment than in a cluttered one.

A prominent theory in visual perception, the Reverse Hierarchy Theory^13^, states that perception first occurs in an automatic feedforward manner, resulting in an almost instantaneous understanding of the visual environment. Relating this theory to appraisal theories^16,44^ and the theories of threat detection and aesthetic pleasure discussed above, we begin to understand why ambiguity is such an important cue of potential threat. An open landscape, for instance, is processed in an automatic fashion and can be parsed quickly because it contains few features, leading to a positive appraisal. This is a reasonably safe environment, because if a snake happens to slither through this environment, it could be rapidly detected and avoided. On the other hand, an environment containing many overlapping and chaotic features would not be understood or processed as quickly. If a snake were to slither through this type of scene, it could go undetected, which would be potentially fatal if we accidentally encroached on its territory and got bitten. Thus, it would serve the early human well to be cautious of such an environment. Assuming that the humans alive today are here because their ancestors were wary of such ambiguous scenes^49^, it makes sense that short angular contours, which may cue these cluttered and chaotic environments, are related to judgements of valence and threat.

Thus far, we have taken an evolutionary interpretation of our results. The evolutionary account seems to be supported by ample evidence^11,12,30^ and has been used to interpret many existing results within this field. However, in the current study, we cannot rule out a learning account, which would suggest that preferences for certain combinations of contour features are learned throughout development. Evidence for the learning account comes from studies of expertise^35^ and familiarity^50^. Since our current study did not assess these factors, we cannot directly evaluate the learning account in our experiments. There is likely a dynamic interplay between learned and evolved processes that should be further elucidated as the research continues. We have shown that contour features are certainly an important aspect of scene perception and emotional or aesthetic judgements, whether learned on innate.

Overall, we have shown that the features important for scene perception (i.e., cues to spatial relationships^15,27^) influence valence and threat judgements in photographs and line drawings. Our work is consistent with work done on isolated objects and geometric shapes^6,7^, and extends the findings to more complex stimulus sets – objects within scenes, landscapes, and abstract drawings made up of combinations of contour features that are known to relate to different scene layouts^15^. By finding similar results in whole scenes, or scene-like images, as have been found on objects, and given that humans only ever encounter whole scenes and objects embedded within scenes (not isolated objects) in daily life, our results potentially suggest that the previous findings in the object domain are a special case of the more general importance of valence and threat cues in complex visual scenes. As mentioned above, if we are close enough to see individual features of a threatening animal, we would not have enough time to protect ourselves from danger. Thus, it may be the case that the cues related to valence and threat are not being fully captured in the size scale of isolated objects or close-up objects without much of the background in view (as is the case in many IAPS images). This could be another interpretation for why the predictive power of the contour features in Experiment 1 was quite low. Indeed, our additional analysis of the Experiment 1 data, splitting the image set into close and far image sets revealed that the contour features better predicted valence ratings for the far images than for the close images, suggesting that the scene context or background may be an important factor. The predictive power of the contours may actually be even higher for solely scene/landscape images without a central object than for objects against a scene background, though more work with a large scene-only image set would be needed to explore this possibility.

One limitation of the current work is that we used abstract line drawings in Experiment 2, which inherently limits the ecological validity of the study. Therefore, although our stimuli are a step-up from simple shapes and isolated objects, future work in this field should certainly aim for even more ecological validity by using more realistic stimulus sets, such as 3D rendered scenes, for example. Fortunately, our approach can be easily extended to other stimulus sets and other sensory domains. Köhler’s (1929) iconic work on shape-speech correspondence^51^, i.e., the ‘Bouba-Kiki’ effect, revealed a systematic link between visual features and their associated sounds, or “names”, and has since been extended to sensory association words such as “sweet” (curved) and “sour” (angular)^52^. This finding suggests that the association of angularity and threat, or other emotional appraisals, could potentially exist in other sensory modalities, if a measurable association between a visual stimulus and another sensory modality exists. Indeed, recent work has found that frequency information available in speech can predict the emotion of a speaker^53^. Another study suggests that fractals at certain spatial scales are also rated as more or less pleasant in the visual^54^ and tactile^55^ domains. A correspondence between curvy and angular shapes to specific aspects of all five senses has also been found^56^. Whether this effect can be found using combinations of features across higher-level stimulus types for all five senses remains an open and unanswered question. Our approach of manipulating combinations of features and measuring their effect on emotional ratings can be used in any sensory domain and stimulus type as long as there is a hypothesis of which sensory features might matter. Combinations of sensory features that show some correspondence to cluttered threatening scenes in the visual domain, such as short bursts of high spatial frequency sounds or a difficult-to-navigate spiky and rocky terrain, for example, would be a good place to start.

The association between angularity and negative emotions has not only been found during passive viewing or listening, but also through action, specifically drawing. When people were prompted to draw shapes to match a given emotion word (e.g., angry), they drew shapes with jagged lines and sharp angles^53^. Of course, angularity is not the only contributing factor in emotional judgements, as we have shown. Future versions of this approach could be used to determine whether a designer or architect who uses manipulations of shape or form in their designs succeeds in communicating the desired emotion through his or her use of shape or form. Adding colour and texture features to the analysis would provide an even more complete picture of the link between visual features and emotion.

Although here we only focused on contour features that are known to be important for rapid scene perception, the potential applications of this approach are far-reaching – from evaluating whether your latest amateur photography collection conveys the desired emotions, to tagging an advertised product with particular emotions, to even using augmented reality on your smartphone’s camera to assess the potential danger of a new environment while travelling abroad. These are all possibilities once we understand which aspects of the visual environment cue valence, threat, and various other emotional appraisals. Our work provides an important step forward in this endeavour – discovering the combinations of physical features that allow people to make affective judgements of visual scenes.

## Methods

### Experiment 1

#### Stimuli

1182 IAPS photographs were used in the analysis. IAPS images are used to study emotion and attention, and include normative ratings of affect for each image. The valence ratings were obtained in 18 separate studies by the researchers who created this image database at the University of Florida^57^. In these studies, participants had to view each image and then rate the image based on how it made them feel. Valence was judged on a 9-point Likert scale from ‘unhappy’ (low ratings) to ‘happy’ (high ratings). The ‘unhappy’ end of the scale referred to feeling unhappy, annoyed, unsatisfied, melancholic, despaired, and/or bored, while the ‘happy’ end of the scale referred to feeling happy, pleased, satisfied, contented, and/or hopeful.

### Feature extraction

The image features for each IAPS image were extracted computationally via line drawings of each image. The line drawings were produced by artists at the Lotus Hill Research Institute (People’s Republic of China) by tracing the original IAPS colour photographs using a custom graphical user interface. The artists were instructed to trace all important lines so that if a human observer were to look at the line drawings, they would be able to recognize the image (i.e., the scene being depicted and the objects within the scene). The resulting line drawings were 800 (width) × 600 (height) pixels in size. Contour properties (orientation, length, and curvature) were computed from the geometrical information of the line drawings. To quantify the distribution of these properties, we computed histograms of each property for every IAPS line drawing. Each property’s histogram was computed in eight bins, each bin corresponding to a particular range of values for that property. See Fig. 1 for an example image and its feature histograms. Calculations of the properties are briefly discussed below. For a more detailed mathematical description of the feature extraction, see Walther and Shen’s (2014) Supplemental Materials^15^. Note that they use the term “Curvature” in a mathematical sense (rate of change of angle per unit length). We here use the term “Angularity”, which is more commonly used to describe the smooth vs. sharp quality of contour lines in images.

The contour properties were calculated on individual lines (i.e., contours) within the line drawing. Contours were defined as a series of fiducial points connected by straight lines, starting with the artist putting down the tip of the graphics pen, and ending with the pen lifted up. The points occur at bends in the contour. Orientation was calculated on each individual line segment within a contour. The orientation of a line segment refers to its counter-clockwise angle from horizontal. The orientation histogram’s bin centers were at 0°, 22.5°, 45°, 67.5°, 90°, 112.5°, 135°, and 157.5°, thus the orientation features ranged from horizontal, to vertical, back to almost horizontal (labeled Orientation 1, Orientation 2, … Orientation 8).

The length feature of the line drawing ranged from short to long (Length 1 to Length 8). The length of each contour was calculated by summing the lengths of the line segments within a contour. The range of lengths of all lines in the image set goes from 1 to 2789 pixels. There is a large proportion of contours on the short end of the range, thus, we equally bin the logarithms of the length measures, resulting in bins with centers at 1.66, 4.47, 12.02, 32.36, 87.10, 232.42, 630.96, and 1698.24 pixels. Across all the images, only 44 out of 1182 (3.7%) contained contours within the range of bin Length 8, thus this feature was dropped from the regression analysis.

Angularity was calculated as the angle difference between adjacent line segments within a contour, divided by the length of the line segments. The angularity features ranged from low to high angularity (i.e., sharp angle changes) (Angularity 1 to Angularity 8). We equally bin the logarithms of the angularity measures, resulting in bins with centers at 1.38, 2.63, 5.13, 9.77, 18.62, 35.48, 67.61, 131.83 degrees per pixel.

### Experiments 2a and 2b

#### Stimuli

In these experiments, we *created* images made up of several contours, taken from 472 line drawings of scene images^15^. The line drawings of the scene images were traced in the same way as the IAPS images were. However, these images were completely independent from the IAPS image set, not strongly emotionally evocative, and only contained images depicting six scene categories (i.e., beaches, forests, mountains, city streets, highways, and offices). Therefore, the newly created abstract images potentially retain ‘scene-like’ properties due to the contours being selected from a scene image set, but have no coherent semantic content.

In order to create the scene-like images with different feature combinations, we first calculated the feature properties for all individual contours from the line drawing scene image set. We calculated the length of each contour, along with the average angularity and average orientation. Average orientation was calculated by first collapsing all orientations to a 90° range (i.e., 180° = 0°), and then taking the arithmetic mean of orientation values within a contour. Each contour was then placed into one of 24 bins, based on the combination of their feature values.

The contours were then randomly sampled one at a time from a particular bin in order to make 20 different images that all contained that particular combination of image features. To create an image, a contour was placed in a location within a 200 × 150-pixel grid. This smaller grid size was chosen in order to accommodate four images on the computer screen at once without the participant having to scroll down or to the side to view the images. The location of each contour was based on its location in the original scene image set, but scaled by a factor of four so that it could fit within the smaller image space. For example, if the contour began at pixel [400, 300] in the original image, its starting location in the new smaller image would be [100, 75]. If the resulting starting location of the contour caused the end of the contour to be outside of the image size limits, the contour was shifted so that it could be accommodated within the 200 × 150-pixel grid in its entirety. If the contour still exceeded the size of the new image after the adjustment, the length of the contour was shortened to accommodate its position within the grid. Contours were added to the image until the total length of the contours exceeded 1000 pixels. Subsequent contours would then be added to a new blank 200 × 150-pixel grid, and so on, until 20 images were created. In the end, 480 images were created (20 per bin) to be used in the experiment (see Fig. 3 for sample images).

### Procedure

A total of 157 people (mean age = 39.1; 61 women) in Experiment 2a and 157 (mean age = 38.4; 62 women) in Experiment 2b participated in an experiment on Amazon Mechanical Turk, and were paid between USD 1.75 and USD 2 for their participation. We recruited MTurk workers with at least a 95% approval rating, and who were located only in the United States or Canada. This study was approved by the University of Toronto Research Ethics Board (Protocol number 30999) and all methods were performed in accordance with the guidelines and regulations of the Ethics Board. All participants provided their informed consent to participate. Power analyses using wp.logistic from the R package WebPower^58^ determined the sample sizes for both Experiments 2a and 2b (*N* = 157) to find a significant main effect of angularity with a moderate effect size (60% chance that high angularity images would be chosen as negative, and 40% chance they would be chosen as positive) at 70% power. The experiment was run using Inquisit software (Version 5.0.14, millisecond.com), and took approximately 20–30 min to complete, including self-paced breaks.

Each participant viewed 4 images per trial, randomly selected from the total 480-image stimulus set, for a total of 120 trials. Each image was presented once during the experiment. Below the images, participants were asked two questions. For Experiment 2a, the questions were: “Which image is the most positive?” and “Which image is the most negative?”. For Experiment 2b, the questions were: “Which image is the most safe?” and “Which images is the most threatening?”. There was a textbox beside each question where participants had to type in their chosen number. Their options were “1”, “2”, “3”, or “4”, each corresponding to one of the displayed images. Following the main experiment, participants filled out demographic information such as age and gender, and responded to the following questions by typing their answer into the response box:

Experiment 2a: 1) “Which features do you typically associate with positive images?”; 2) “Which features do you typically associate with negative images?”; and 3) “Any other comments about how you made your positive/negative judgements:”.

Experiment 2b: 1) “Which features do you typically associate with safe images?”; 2) “Which features do you typically associate with threatening images?”; and 3) “Any other comments about how you made your safe/threatening judgements:”.

The data from Experiments 1, 2a, and 2b, and the stimuli from Experiment 2 are available on OSF framework, and can be downloaded at the following link: https://osf.io/y3rjm/.

## References

[1] Ellard CG, Eller MC (2009). Spatial cognition in the gerbil: computing optimal escape routes from visual threats. Anim. Cogn..

[2] LoBue V, DeLoache JS (2010). Superior detection of threat-relevant stimuli in infancy: threat detection in infancy. Dev. Sci..

[3] Mobbs D, Hagan CC, Dalgleish T, Silston B, Prévost C (2015). The ecology of human fear: survival optimization and the nervous system. Front. Neurosci..

[4] Gao X, LoBue V, Irving J, Harvey T (2017). The effect of spatial frequency information and visual similarity in threat detection. Cogn. Emot..

[5] LoBue V (2014). Deconstructing the snake: the relative roles of perception, cognition, and emotion on threat detection. Emotion.

[6] Bar M, Neta M (2006). Humans prefer curved visual objects. Psychol. Sci..

[7] Larson CL, Aronoff J, Stearns JJ (2007). The shape of threat: simple geometric forms evoke rapid and sustained capture of attention. Emotion.

[8] Aronoff J, Barclay AM, Stevenson LA (1988). The recognition of threatening facial stimuli..

[9] Bar M, Neta M (2007). Visual elements of subjective preference modulate amygdala activation. Neuropsychologia.

[10] Larson CL, Aronoff J, Sarinopoulos IC, Zhu DC (2009). Recognizing threat: a simple geometric shape activates neural circuitry for threat detection. J. Cogn. Neurosci..

[11] Munar E, Gómez-Puerto G, Call J, Nadal M (2015). Common visual preference for curved contours in humans and great apes. PloS One.

[12] Fantz, R. L., & Miranda, S. B. (1975). Newborn infant attention to form of contour. *Child Development*, 224–228. 1132272

[13] Hochstein S, Ahissar M (2002). View from the top: Hierarchies and reverse hierarchies in the visual system. Neuron.

[14] Oliva A, Torralba A (2006). Building the gist of a scene: The role of global image features in recognition. Prog. Brain Res..

[15] Walther DB, Shen D (2014). Nonaccidental properties underlie human categorization of complex natural scenes. Psychol. Sci..

[16] Scherer KR (1999). On the sequential nature of appraisal processes: indirect evidence from a recognition task. Cogn. Emot..

[17] Susskind JM, Lee DH, Cusi A, Feiman R, Grabski W, Anderson AK (2008). Expressing fear enhances sensory acquisition. Nat. Neurosci..

[18] Cheng, A., Walther, D. B., Park, S., & Dilks, D. D. (2021). Concavity as a diagnostic feature of visual scenes. *NeuroImage, 232*, 117920. 10.1016/j.neuroimage.2021.117920 PMC8256888 33652147

[19] Vartanian O, Navarrete G, Chatterjee A, Fich LB, Leder H, Modroño C, Skov M (2013). Impact of contour on aesthetic judgments and approach-avoidance decisions in architecture. Proc. Natl. Acad. Sci..

[20] Ruta N, Mastandrea S, Penacchio O, Lamaddalena S, Bove G (2019). A comparison between preference judgments of curvature and sharpness in architectural façades. Archit. Sci. Rev..

[21] Lakens D, Fockenberg DA, Lemmens KPH, Ham J, Midden CJH (2013). Brightness differences influence the evaluation of affective pictures. Cogn. Emot..

[22] Cano, M. E., Class, Q. A., & Polich, J. (2009). Affective valence, stimulus attributes, and P300: Color vs. black/white and normal vs. scrambled images. *Int. J. Psychophysiol.*, *71*(1), 17–24. 10.1016/j.ijpsycho.2008.07.016 10.1016/j.ijpsycho.2008.07.016 PMC2664687 18708099

[23] Delplanque S, N’diaye, K., Scherer, K., & Grandjean, D. (2007). Spatial frequencies or emotional effects?. J. Neurosci. Methods.

[24] Redies C, Grebenkina M, Mohseni M, Kaduhm A, Dobel C (2020). Global image properties predict ratings of affective pictures. Front. Psychol..

[25] Walther DB, Chai B, Caddigan E, Beck DM, Fei-Fei L (2011). Simple line drawings suffice for functional MRI decoding of natural scene categories. Proc. Natl. Acad. Sci..

[26] Choo H, Walther DB (2016). Contour junctions underlie neural representations of scene categories in high-level human visual cortex. Neuroimage.

[27] Wilder J, Rezanejad M, Dickinson S, Siddiqi K, Jepson A, Walther DB (2019). Local contour symmetry facilitates scene categorization. Cognition.

[28] Berman, D., Golomb, J. D., & Walther, D. B. (2017). Scene content is predominantly conveyed by high spatial frequencies in scene-selective visual cortex. *PLoS One, 12*(12), e0189828. 10.1371/journal.pone.0189828 PMC5741213 29272283

[29] Perfetto S, Wilder J, Walther DB (2020). Effects of spatial frequency filtering choices on the perception of filtered images. Vision.

[30] Gómez-Puerto G, Munar E, Nadal M (2016). Preference for curvature: a historical and conceptual framework. Front. Hum. Neurosci..

[31] Palumbo L, Ruta N, Bertamini M (2015). Comparing Angular and Curved Shapes in Terms of Implicit Associations and Approach/Avoidance Responses. PLoS ONE.

[32] Bates D, Maechler M, Bolker B, Walker S (2015). Fitting linear mixed-effects models using lme4. J. Stat. Softw..

[33] R Core Team (2019). R: A language and environment for statistical computing. R Foundation for Statistical Computing, Vienna, Austria. https://www.R-project.org/.

[34] Singmann, H., Bolker, B., Westfall, J., Aust, F., & Ben-Shachar, M.S. (2020). afex: Analysis of Factorial Experiments. R package version 0.26–0. https://CRAN.R-project.org/package=afex

[35] Silvia PJ, Barona CM (2009). Do people prefer curved objects? angularity, expertise, and aesthetic preference. Empir. Stud. Arts.

[36] Isbell LA (2006). Snakes as agents of evolutionary change in primate brains. J. Hum. Evol..

[37] Van Le Q, Isbell LA, Matsumoto J, Nguyen M, Hori E, Maior RS, Nishijo H (2013). Pulvinar neurons reveal neurobiological evidence of past selection for rapid detection of snakes. Proc. Natl. Acad. Sci..

[38] Jakesch, M., Leder, H., & Forster, M. (2013). Image ambiguity and fluency. *PLoS One*, *8*(9), e74084. 10.1371/journal.pone.0074084 PMC3764012 24040172

[39] Reber R, Schwarz N, Winkielman P (2004). Processing fluency and aesthetic pleasure: is beauty in the perceiver's processing experience?. Pers. Soc. Psychol. Rev..

[40] Van Geert E, Wagemans J (2020). Order, complexity, and aesthetic appreciation. Psychol. Aesthet. Creat. Arts.

[41] Damiano, C., Wilder, J., Zhou, E. Y., Walther, D. B., & Wagemans, J. (2021). The role of local and global symmetry in pleasure, interest, and complexity judgments of natural scenes. *Psychology of Aesthetics, Creativity, and the Arts*.

[42] Kaplan, R., & Kaplan, S. (1989). *The experience of nature: A psychological perspective*. Cambridge university press.

[43] Ulrich RS, Simons RF, Losito BD, Fiorito E, Miles MA, Zelson M (1991). Stress recovery during exposure to natural and urban environments. J. Environ. Psychol..

[44] Moors A, Ellsworth PC, Scherer KR, Frijda NH (2013). Appraisal theories of emotion: State of the art and future development. Emot. Rev..

[45] Biederman, I., & Vessel, E. (2006). Perceptual Pleasure and the Brain: A novel theory explains why the brain craves information and seeks it through the senses. *American Scientist*, 8.

[46] Ibarra FF, Kardan O, Hunter MR, Kotabe HP, Meyer FA, Berman MG (2017). Image feature types and their predictions of aesthetic preference and naturalness. Front. Psychol..

[47] Herzog TR, Chernick KK (2000). Tranquility and danger in urban and natural settings. J. Environ. Psychol..

[48] Ross MG, Oliva A (2010). Estimating perception of scene layout properties from global image features. J. Vis..

[49] Whalen PJ (1998). Fear, vigilance, and ambiguity: Initial neuroimaging studies of the human amygdala. Curr. Dir. Psychol. Sci..

[50] Leder H, Carbon CC (2005). Dimensions in appreciation of car interior design. Appl. Cognit. Psychol..

[51] Köhler W (1929). Gestalt psychology.

[52] Salgado-Montejo A, Alvarado JA, Velasco C, Salgado CJ, Hasse K, Spence C (2015). The sweetest thing: the influence of angularity, symmetry, and the number of elements on shape-valence and shape-taste matches. Front. Psychol..

[53] Sievers B, Lee C, Haslett W, Wheatley T (2019). A multi-sensory code for emotional arousal. Proc. R. Soc. B.

[54] Spehar B, Clifford CW, Newell BR, Taylor RP (2003). Universal aesthetic of fractals. Comput. Graph..

[55] Viengkham, C., Isherwood, Z., & Spehar, B. (2019). Fractal-scaling properties as aesthetic primitives in vision and touch. *Axiomathes*, 1–20.

[56] Blazhenkova O, Kumar MM (2018). Angular versus curved shapes: Correspondences and emotional processing. Perception.

[57] Lang, P. J., Bradley, M. M., & Cuthbert, B. N. (2008). *International affective picture system (IAPS): affective ratings of pictures and instruction manual. University of Florida, Gainesville*. Tech Rep A-8.

[58] Zhang, Z., & Yuan, K.-H. (2018). Practical Statistical Power Analysis Using Webpower and R (Eds). Granger, IN: ISDSA Press.

